# Electrochemistry of Flavonoids

**DOI:** 10.3390/molecules28227618

**Published:** 2023-11-16

**Authors:** Dorota Naróg, Andrzej Sobkowiak

**Affiliations:** Department of Physical Chemistry, Faculty of Chemistry, Rzeszów University of Technology, 35-959 Rzeszów, Poland

**Keywords:** flavonoids, electrochemical oxidation, redox properties, cyclic voltammetry

## Abstract

This review presents a description of the available data from the literature on the electrochemical properties of flavonoids. The emphasis has been placed on the mechanism of oxidation processes and an attempt was made to find a general relation between the observed reaction paths and the structure of flavonoids. Regardless of the solvent used, three potential regions related to flavonoid structures are characteristic of the occurrence of their electrochemical oxidation. The potential values depend on the solvent used. In the less positive potential region, flavonoids, which have an *ortho* dihydroxy moiety, are reversibly oxidized to corresponding *o*-quinones. The *o*-quinones, if they possess a C3 hydroxyl group, react with water to form a benzofuranone derivative (**II**). In the second potential region, (**II**) is irreversibly oxidized. In this potential region, some flavonoids without an *ortho* dihydroxy moiety can also be oxidized to the corresponding *p*-quinone methides. The oxidation of the hydroxyl groups located in ring A, which are not in the *ortho* position, occurs in the third potential region at the most positive values. Some discrepancies in the reported reaction mechanisms have been indicated, and this is a good starting point for further investigations.

## 1. Introduction

**Scheme 1:**
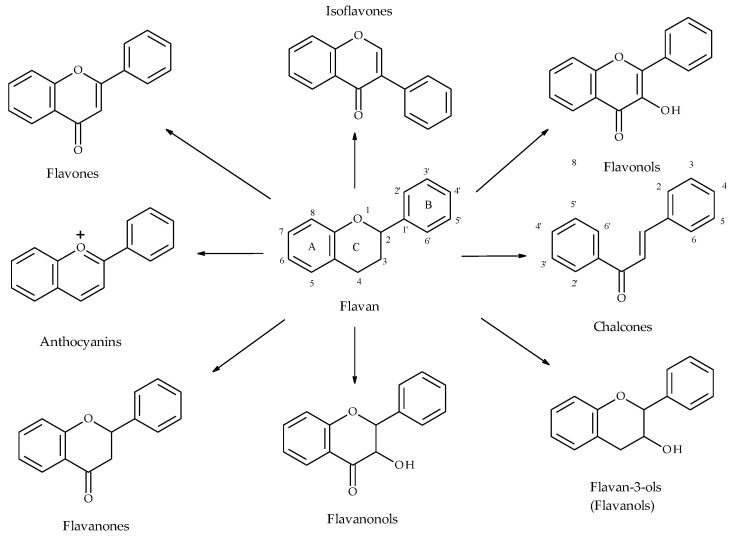
Chemical structures of the most important classes of flavonoids.

Flavonoids are polyphenolic compounds synthesized as bioactive secondary metabolites, commonly found in fruits, vegetables, and certain beverages (tea, coffee, chocolate, and red wine). Chemically, a flavonoid molecule consists of two aromatic rings (A and B) and a heterocyclic 6-atomic ring (C) containing the ether group. Flavan (2-phenyl-3,4-dihydro-2*H*-1-benzopyran) is considered a basic skeleton of the flavonoid structures [1]. The main difference between flavonoids, which is the basis for their classification, is the existence of a double C2–C3 bond and a carbonyl group at C4. Scheme 1 presents the most widespread classes of flavonoids: flavones, flavonols, flavanones, flavanonols, isoflavones, flavan-3-ols, anthocyanins, and chalcones.

The vast number of known flavonoids (approximately 10,000) is due to the possibility of the presence of three types of substituents (hydroxy, methoxy, and glycosyl) in different positions [2,3,4,5,6]. The presence of hydroxyl groups, and to some extent, methoxy groups, makes flavonoids suitable for oxidation. In plants, flavonoids occur mainly in the form of *O*-glycosides [7,8], in which a sugar moiety is connected to the flavonoid molecule through an oxygen atom in position C3 or C7. Recently, many *C*-glycosylflavonoids [9,10], whose glycosyl moiety is attached by the anomeric carbon directly to the flavonoid backbone, usually in position C6 or C8, have been isolated and identified from a wide variety of plant species.

Flavonoids are beneficial for human health with their antiviral, antibacterial, antifungal, anti-inflammatory, and anticancer activities, as well as cardiovascular protection [3,4,5]. Recently, a hypothesis about the role of flavonoids in the process of inhibiting the action of key proteins involved in the coronavirus infection cycle has been presented [11]. These activities of flavonoids are related to their chelating and antioxidant properties. Flavonoids play an important role in metal chelating (iron, copper, etc.) and therefore prevent the generation of oxygen-containing free radicals in the oxygen metabolism process [3,12,13]. The chelating properties of flavonoids, which were also confirmed by a single crystal X-ray analysis [14,15], are the result of the presence of hydroxyl groups in *ortho* positions or the carbonyl group and a hydroxyl group in position C3 or C5.

**Scheme 2:**
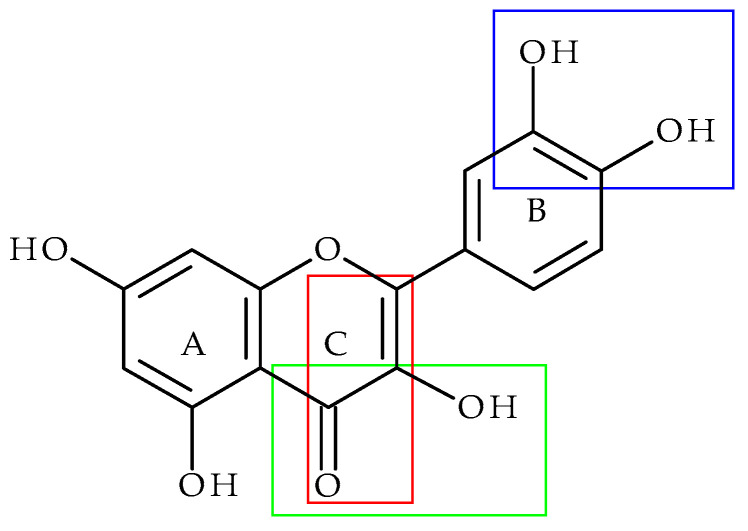
The main structural features of flavonoids responsible for their antioxidative properties, based on the quercetin molecule.

However, the main activity of flavonoids is their antioxidant activity, which can prevent damage caused by free radicals by scavenging reactive species, activating antioxidant enzymes, inhibiting oxidases, and reducing *α*-tocopheryl radicals [6,12,16,17]. Generally, the antioxidative properties of flavonoids depend on the presence of a catechol moiety in ring B, the C2–C3 double bond conjugated with the C4-oxo group in ring C, and the C3-hydroxyl group, which is shown in Scheme 2 on the example of a quercetin (3,3′,4′,5,7-pentahydroxyflavone) molecule.

It has been shown [18] that the antioxidative impact of flavonoids on human health is related to their redox activities. Therefore, these compounds have been the target of numerous electrochemical investigations since knowledge of their electrooxidation mechanism can help to elucidate the mechanism of the antioxidant action. On the other hand, the electrochemical activity of flavonoids has been utilized for electroanalytical applications [19] and sensor constructions [20,21]. Recent reviews describe materials for electrode construction [22] and electrochemical techniques [23], which are most commonly used for the electrochemical determination of the concentration of flavonoids. However, in this review, a description of available data in the literature on the electrochemistry of flavonoids with an emphasis on the mechanisms of their transformations is presented. We have tried to systematize the reported data and find general rules that show the dependence of the mechanism of the electrochemical oxidation of flavonoids on their structure.

## 2. The Electrochemistry of Flavonoids in Water-Based Solutions

The electrochemical behaviour of flavonoids has been studied mainly in mixed water-organic solvents because of their relatively low solubility in water. Although most articles are dedicated to oxidation processes, one of the first studies on flavonoid electrochemistry deals with the reduction of quercetin and its derivatives as well as apigenin (4′,5,7-trihydroxyflavone) and flavonol (3-hydroxyflavone) on dropping mercury electrode in aqueous buffer solutions containing 50% isopropyl alcohol [24]. The presence of polarographic waves, with half-wave potentials between −1.16 and −1.63 V (vs. SCE) at pH 7.7 was observed. It has been suggested that the reduction in the carbonyl group occurs and the possibility of hydrogen bond formation between the carbonyl group and the C5 hydroxyl group is responsible for the shift of the half-wave potential toward more negative values. In a subsequent paper [25], the polarographic reduction in some substituted chalcones, flavones, and flavanones in the same experimental conditions has been described.

In one of the early papers on flavonoid electrochemical oxidation, the first oxidation potentials of a series of structurally related flavonoids measured by cyclic voltammetry on a glassy carbon electrode (GCE) in a phosphate buffer containing 2.5% dimethyl sulfoxide were reported [26]. It has been found that the presence of an *o*-dihydroxy moiety in ring B of a flavonoid molecule is responsible for two-electron reversible oxidation, but the reversibility decreases with the decrease in the scan rate. During the oxidation process, the presence of semiquinone radicals was detected by an electron paramagnetic resonance spectroscopy (EPR), which indicates that a radical is formed in the first step of the process. It was also shown that the oxidation products were adsorbed on the electrode. The importance of the presence of the *o*-dihydroxy moiety in the existence of reversible oxidation has been shown by the comparison of the cyclo voltammetric behaviour of rutin (quercetin 3-rutinoside), which contains the *o*-dihydroxy moiety in ring B and kaempferol 3-rutinoside without the moiety [27]. Rutin exhibits two-electron reversible oxidation at the potential, which is approximately 0.5 V more positive than the potential at which irreversible, one-electron oxidation of kaempferol 3-rutinoside occurs.

Cyclic voltammetric measurements in the wider potential range of structurally related flavonoids: quercetin, quercitrin (quercetin-3-rhamnoside), rutin, and luteolin (3′,4′,5,7-tetrahydroxyflavone) have shown the existence of up to three oxidation peaks [28]. The structures of the flavonoids investigated are similar to the quercetin structure; however, in the case of luteolin there is no hydroxyl group at C3 and for quercitrin and rutin, the C3 hydroxyl group is blocked by a sugar moiety. The first reversible oxidation is caused by the presence of two hydroxy substituents present in the B ring in the *ortho* position (3′,4′), leading to the formation of quinone. The existence of the second irreversible oxidation peak is related to the presence of the hydroxyl group at C3. This oxidation is not observed for quercitrin, rutin, or luteolin where the C3 hydroxyl group is conjugated to a sugar or is absent. The final irreversible oxidation is due to the presence of a hydroxyl group in the A ring. The last conclusion was supported by the comparison of the observed potentials of the third peak with those obtained for 3-hydroxyflavone, 7-hydroxyflavone, chrysin (5,7-dihydroxyflavone), and galangin (3,5,7-trihydroxyflavone). As the scan rate decreases, the reversibility of the first redox couple also decreases. The presence of the C3 hydroxyl group (quercetin) has a greater influence on the relationship between reversibility and scan rate. The observed influence of the scan rate on the degree of irreversibility indicates that the products of electrochemical oxidation undergo further chemical reactions. An attempt was made to determine the products of the reactions.

In several subsequent papers [29,30,31,32] devoted to the electrochemical oxidation of quercetin, there was common agreement that the first oxidation process is related to the formation of quercetin 3′,4′-quinone. Except for consideration on the following oxidation product [29,30], it was indicated that the *o*-quinone formed in the first step reacts with benzenesulfinic acids to form the corresponding sulfonyl derivatives [31], and electrochemical parameters such as the heterogeneous charge transfer rate constant, transfer coefficient, exchange current density, and also diffusion coefficient of quercetin were calculated [32].

The electrochemical behaviour of quercetin on a glassy carbon electrode in the mixed water-ethanol matrix within the pH range of 2.2 to 9.2 was studied by cyclic voltammetry and controlled potential electrolysis followed by UV-vis spectroscopy [33]. It has been shown that an increase in pH causes a shift in the first oxidation peak toward more negative values and the dependence is linear. The increase in pH and the increase in the scan rate decrease the reversibility of the process. The decrease in the reversibility of the oxidation process on scan rate indicates that a product of electron/proton transfer undergoes a chemical reaction.

**Scheme 3:**
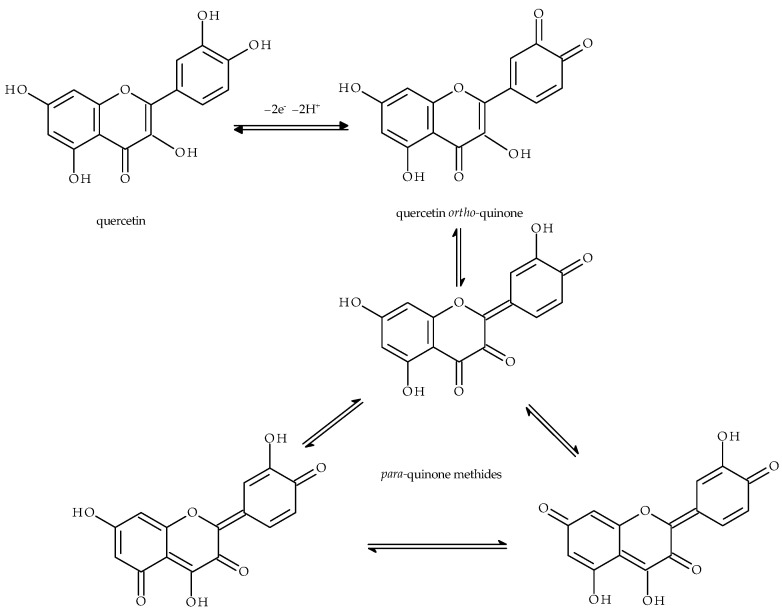
Quinone species formed during the electrochemical oxidation of quercetin at the first oxidation potential. Adopted with permission from Ref. [33]. Copyright 2006, Sociedade Brasileira de Química.

**Scheme 4:**
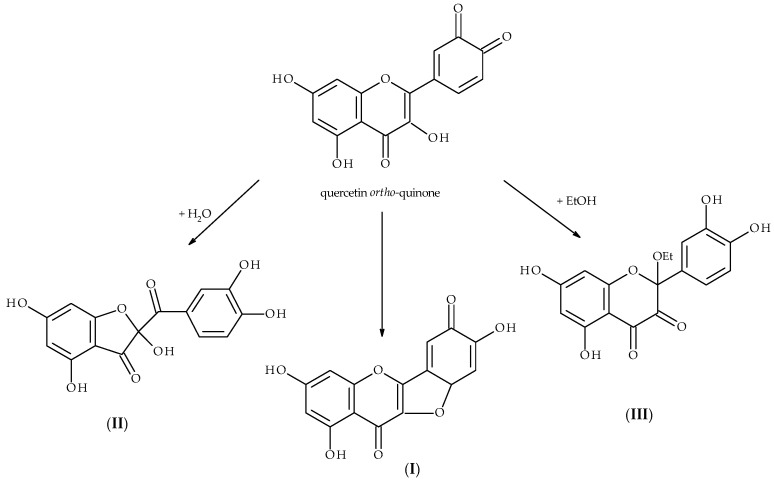
Possible follow-up reactions after the electrochemical oxidation of quercetin in the water-ethanol matrix at the first oxidation potential. Adopted with permission from Ref. [33]. Copyright 2006, Sociedade Brasileira de Química. Copyright 2006, Sociedade Brasileira de Química.

Based on the result of the bulk electrolysis connected with the UV-vis spectra of the products, the authors concluded that quercetin-*o*-quinone, or its tautomeric forms (*p*-quinone methide, Scheme 3) can undergo intramolecular rearrangement, react with water or alcohol to give as products: (**I**)—1,3,8-trihydroxy-11H-benzofuro[3,2-b][1]benzopyran-7,11(9aH)-dione, (**II**)—2-(3′,4′-dihydroxybenzoyl)-2,4,6-trihydroxybenzofuran-3(2H)-one, and (**III**)—2-(3′,4′-dihydroxyphenyl)-2-ethoxy-5,7-dihydroxy-chromane-3,4-dione respectively, presented in Scheme 4. 

In conclusion, it was postulated that the oxidation processes at the second and third peaks do not correspond to the oxidation of the hydroxyl group present at C3, and also at C5 and C7, respectively, as previously suggested, but can be ascribed to the oxidation of the above-mentioned products. However, the statement related to the third peak seems unjustified. The previous study [28] has shown that there is a peak in this potential region during the electrochemical oxidation of flavonoids, which do not have an *o*-dihydroxy moiety (7-hydroxyflavone, 5,7-dihydroxyflavone, and 3,5,7-trihydroxyflavone).

The existence of three peaks during quercetin oxidation and two in the case of rutin was confirmed by cyclic voltammetry on a glassy carbon electrode in phosphate buffer containing 5% ethanol [34]. In this paper, cyclic voltammograms of other natural phenolic compounds were reported, and an attempt was made to correlate their first oxidation potential with antioxidant activities. The three oxidation peaks of quercetin were also observed in an acidic medium on a graphite wax electrode [35]. Using spectroelectrochemical measurements, it was found that the product formed at the first oxidation potential is the 3′,4′-quinone, the subsequent anodic peaks showed an adsorption character and were attributed to further oxidations of intermediate products. Analogues behaviour was reported for the electrochemical oxidation of rutin. However, only two oxidation peaks were present as previously reported [34], the reversible one related to the formation of rutin-3′,4′-quinone, and the irreversible resulting from the oxidation of hydroxyl groups in ring A [36,37,38]. The first peak was also used to determine the concentration of rutin by differential pulse voltammetry [39].

The electrochemical behaviour of (+)-catechin (3,3′,4′,5,7-pentahydroxyflavane), quercetin, and rutin [40], as well as (±)-taxifolin (3,3′,4′,5,7-pentahydroxyflavanone, dihydroquercetin) [41] on a glassy carbon electrode at different pH values, studied by cyclic, differential pulse, and square wave voltammetry has been described. All compounds possess the *o*-dihydroxy moiety in ring B and therefore, as expected, exhibit reversible oxidation peaks at similar potentials, which are connected with *o*-quinone formation. The second peak present for quercetin oxidation at more positive potentials was absent in the case of rutin (the C3 hydroxyl group is blocked by a sugar moiety) and taxifolin (the absence of the C2–C3 double bond). Surprisingly, catechin, which does not have the carbonyl group and, more importantly, the C2–C3 double bond, expresses the distinctive, irreversible oxidation peak in the potential region. The same observation was reported in aqueous [42,43] and nonaqueous [44] media. It was suggested [42] that oxidation of the hydroxyl group at C3 is responsible for the peak formation (by analogy to 3-hydroxyflavone [45]) but no mechanistic details are provided. However, given recent findings [46], the oxidation of 3-hydroxyflavone in non-aqueous media can produce a *p*-quinone methide derivative, and the presence of the C2–C3 double bond is necessary for the process to occur.

Cyclic voltammograms of quercetin and luteolin on glassy carbon and platinum electrodes in different water solutions, without and in the presence of β-cyclodextrin, have been reported [47]. It was shown that the bulk electrolysis of quercetin gives compound (**II**) as the main product, and the presence of β-cyclodextrin increases the product yields.

It is obvious that for the determination of the oxidation mechanism, information about the product formed is necessary. The first attempt in a water-based solution (ethanol-PBS buffer at pH 7.4, 1:1, *v*/*v*) using other than UV-vis spectroscopy techniques for product characterization presents the study, in which quercetin electrooxidation was performed by controlled potential bulk electrolysis on a glassy carbon electrode [48]. The applied potential was equal to +1.0 V (vs. Ag/AgCl, 3 M NaCl), which is approximately 0.9 V more positive than the potential of the first peak of quercetin oxidation measured by cyclic voltammetry. The products were separated by column chromatography and analysed by GC-MS and LC-MS. A total of 18 products were detected, among them compounds (**II**) and (**III**) suggested in [33] (Scheme 4), and also several products resulting from ring-opening and further degradation. The subsequent article [49] compares the products formed in the quercetin oxidation process during bulk electrolysis, autoxidation (air oxygen), enzymatic oxidation (mushroom tyrosinase), and oxidation with azodiisobutyronitrile (AIBN). The large number of oxidation products of the electrooxidation process can be caused by the use of a relatively large oxidation potential. In an earlier study [50] during the bulk electrolysis of quercetin performed in acetonitrile at a potential of 0.2 V more positive than the first peak potential determined by cyclic voltammetry, only compound (**II**) was isolated by HPLC and characterized by ^1^H and ^13^C-NMR spectroscopy. However, the similarity between the quercetin oxidation products obtained during the preparative electrolysis and autoxidation processes is striking [49]. This suggests that molecular oxygen, if present in the reaction environment, can react with the intermediates formed during electrolysis, and therefore be responsible for the large amounts of products observed. The instability of quercetin and luteolin in atmospheric oxygen, especially in alkaline media, leading to several degradation products has also been described in [51]. Compound (**II**) was found among the products of quercetin oxidation, whereas no corresponding benzofuranone derivative of luteolin was detected. The electrochemical oxidation of quercetin, as well as chrysin, flavonol, kaempferol (4′,3,5,7-tetrahydroxyflavone), morin (2′,4′,3,5,7-pentahydroxyflavone), and myricetin (3′,4′,5′,3,5,7-hexahydroxyflavone), on glassy carbon as a working electrode in a methanol-water matrix and in methanol was investigated in an electrochemical flow cell coupled with an electrospray ionization mass spectrometer to identify the products formed [52]. For each investigated flavonoid several products, also methylated derivatives, were detected. Only in the mixed solvent, compound (**I**) was detected during quercetin electrooxidation. However, no benzofuranone derivatives were detected in all cases.

**Scheme 5:**
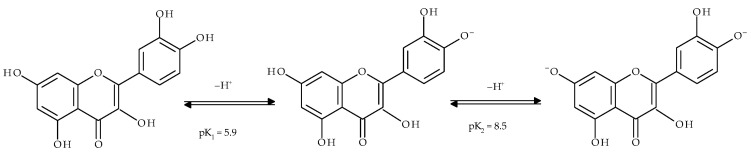
The acid-base equilibria of quercetin, and the values of pKs are taken from Ref. [51].

**Figure 1:**
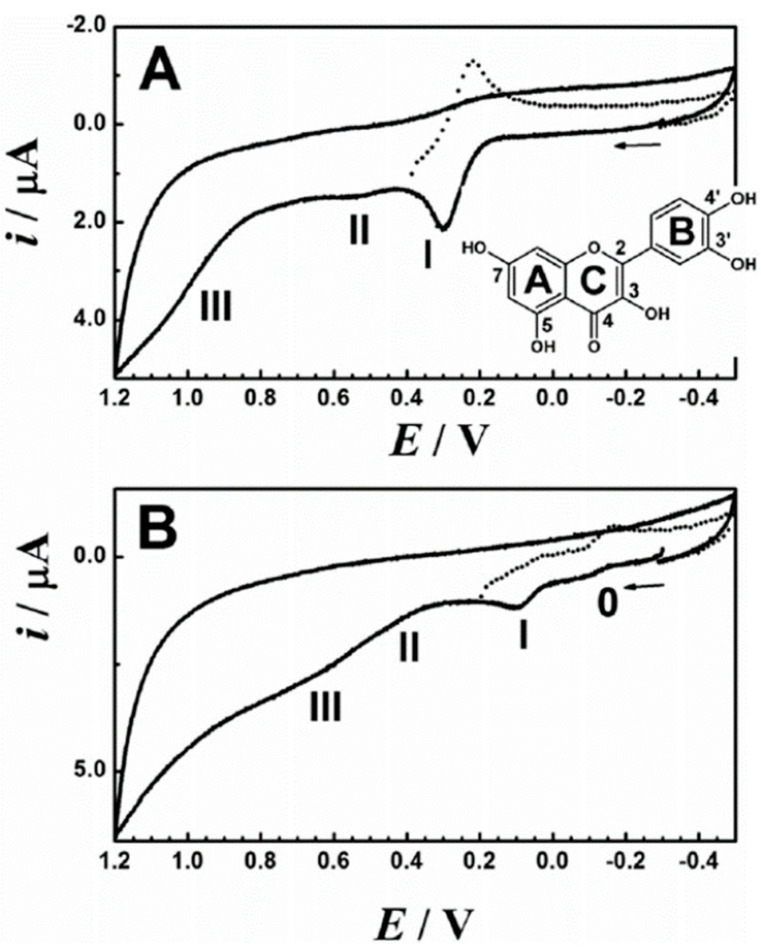
Cyclic voltammogram of 4 × 10^−4^ M quercetin in Britton-Robinson buffer on a glassy carbon electrode at different pH: (**A**) 5.3, (**B**) 10.5. The scan rate is 0.25 V s^−1^. The dotted line represents the cyclic voltammogram when the polarity was changed behind the oxidation peak I. As follows from the Supplementary Materials the reference electrode was Ag/AgCl, 1 M LiCl, which is approximately equal to 0 V vs. SCE. Reproduced with permission from Ref. [53]. Copyright 2012, The Royal Society of Chemistry.

Sokolová et al. [53] presented a detailed study on the electrochemical oxidation of quercetin in Britton–Robinson buffer on a glassy carbon electrode in a wide range of pH [53]. Except for three peaks, reported previously [33], among them, the first, reversible is pH dependent; an additional prepeak has been reported in strong alkaline media at the less positive potential (Figure 1). The prepeak was assigned to the oxidation of quercetin dianion, whereas the oxidation of undissociated quercetin or its monoanion occurs in the first potential region. Scheme 5 presents the acid-base equilibria of quercetin. Furthermore, using spectroelectrochemical measurements, it was proved that the oxidation of 2-(3′,4′-dihydroxybenzoyl)-2,4,6-trihydroxy-benzofuran-3-one (**II**), which is formed in the reaction of quercetin 3′,4′-quinone with water (Scheme 4), is responsible for the appearance of the second peak. The conclusion was made based on previous results [54] on the electrochemical oxidation of quercetin in nonaqueous media, in which (**II**) was identified by HPLC-DAD, GC-MS, and LC-MS techniques as the final oxidation product at the first oxidation peak (assuming the presence of traces of water in the reaction media) and characterized by UV-vis spectroscopy. In a water-based solution [53], (**II**) and a dimmer were the main products after the bulk electrolysis in the absence of dioxygen. However, the products are air-sensitive, and 3,4-dihydroxybenzoic acid was formed as the main stable product during the quercetin electrooxidation in the presence of air. 

**Scheme 6:**
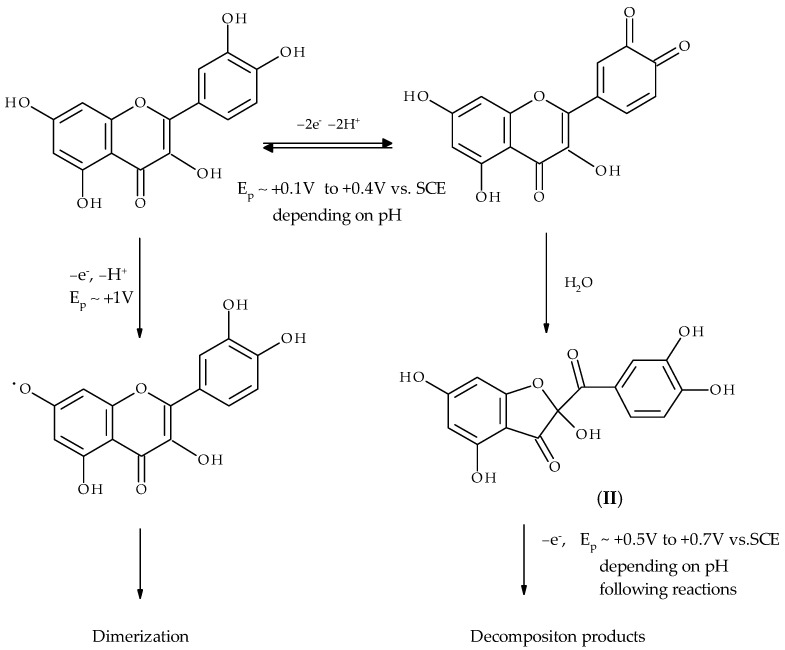
The observed pathways of electrochemical oxidation of quercetin in water media.

Based on the results discussed previously, the general scheme of quercetin oxidation in water media is presented in Scheme 6. The first oxidation process is a reversible oxidation of quercetin (or its dissociated forms) to the corresponding 3′,4′-quinone. The resulting quinone with water forms the benzofuranone derivative—compound (**II**), which undergoes further electrochemical oxidation giving the second peak. The third oxidation peak is associated with the oxidation of an isolated hydroxyl group in ring A. The values of the potentials are pH dependent, they are shifted toward more positive potentials with increasing pH.

The subsequent papers published by the same authors compared the electrochemical behaviour (investigated under similar conditions) of flavonoids structurally closely related to quercetin. The results obtained confirmed the proposed oxidation mechanism. Fisetin [55] (3′,4′,3,7-tetrahydroxyflavone) and rhamnetin [56] (3′,4′,3,5-tetrahydroxy-7-methoxyflavone), which differ from the quercetin molecule by the lack of 5-hydroxyl group (fisetin) and the presence of the methoxy group instead the hydroxyl group at C7 (rhamnetin) show the same voltammetric behaviour as quercetin. This indicates that the arrangement of hydroxyl groups in rings B and C is responsible for the formation of the first two oxidation peaks. 

In the case of rhamnazin [57] (4′,3,5-trihydroxy-the 3′,7-dimethoxyflavone), in which 3′ and 7 hydroxyl groups in the quercetin molecule are replaced by methoxy groups and therefore there is no *o*-dihydroxy moiety, the first reversible peak and the second irreversible peak are shifted toward more positive potentials. It has been shown that the corresponding *p*-quinone methide is formed in the first step. It can give a derivative of benzofuranone, the oxidation of which occurs in the second step. However, it should be noted that the second peak in all cases is not well developed. The third peak exists in the same potential region for all of the discussed compounds, and the presence of a hydroxyl group in any position of ring A is responsible for its formation. In strong alkaline media, the prepeak observed for quercetin and rhamnetin does not appear for rhamnazin. For all of these flavonoids, the corresponding benzofuranone derivatives were found to be the main electrooxidation products, and further degradation products were determined. The cyclic voltammogram of isorhamnetin (4′,3,5,7-tetrahydroxy-3′-methoxyflavone), which likewise rhamnazin has 3′-methoxy and 4′-hydroxy moieties in ring B, recorded on a glassy carbon electrode in a PBS buffer at pH 4, does not show the second peak [58]. However, on a voltammogram of isorhamnetin measured on a glassy carbon electrode in Britton–Robinson buffer containing KCl at pH 7.0, three oxidation peaks were present [59] (see also Table 1, entry 15).

In another report [60] on fisetin voltammetry, a different oxidation mechanism has been proposed based on the assumption that the fisetin *o*-quinone formed in the first step is rearranged to the hydroxyl derivative of compound (**I**), which is further oxidized to the corresponding *o*-quinone. However, the assumption was not confirmed by independent product analysis. The role of fisetin adsorption on the electrode during its electrochemical oxidation has also been indicated [61].

In the case of kaempferol [29,62,63] and morin [62,64,65], which differ from quercetin by the lack of the *o*-dihydroxy moiety in ring B, the first oxidation potential is shifted toward more positive values than that for quercetin. It is a general agreement that the peak results from the corresponding derivative of the *p*-quinone methide formation. The preparative electrolyses have shown that the corresponding benzofuranone derivatives are formed [50,66]. However, separate peaks of the oxidation of the benzofuranone derivatives were not present on the voltammograms in either case. This could indicate that the formation of the *p*-quinone methide and the oxidation of the benzofuranone derivative occurs in the same potential region. The second peak at much more positive potentials is caused by the oxidation of a hydroxyl group present in ring A.

Luteolin differs from quercetin by the lack of the C3 hydroxyl group. Therefore, as expected, a pH-dependent reversible oxidation of the *o*-dihydroxy moiety and the lack of the benzofuranone derivative oxidation follow from the published voltammetric curves [67,68]. The results of the bulk electrolysis of luteolin confirm the absence of the benzofuranone derivative among the products [66,68].

**Scheme 7:**
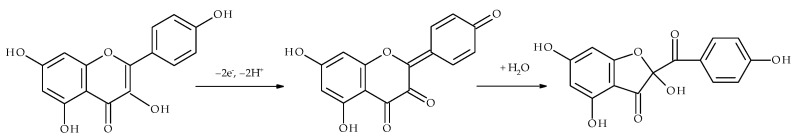
Formation of the benzofuranone derivative during electrooxidation of kaempferol. On the basis of the results presented in Ref. [50].

Taking into account the above considerations, it can be concluded that a benzofuranone derivative is also produced if a flavonoid molecule can form a *p*-quinone methide, i.e., it contains C3 and C4′ hydroxyl groups and the C2–C3 double bond (Scheme 7). 

The same pattern of product formation was also observed for the oxidation of quercetin, fisetin, luteolin, and taxifolin with air in a slightly alkaline solution under ambient conditions [69]. Oxidation of quercetin and fisetin causes the formation of a benzofuranone derivative and several open-ring compounds. In the case of luteolin and taxifolin, the benzofuranone derivative is not formed. These compounds were oxidized to their hydroxylated derivatives and open-ring compounds. It should be noted that the presence of the benzofuranone derivative among quercetin oxidation products has been shown to enhance its antioxidant power [70,71].

**Scheme 8:**
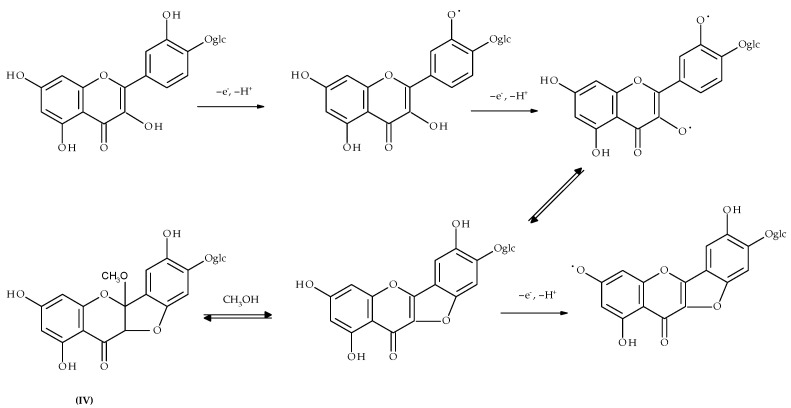
Proposed mechanism for the electrooxidation of quercetin 4′-glucoside. Adapted with permission from Ref. in [72]. Copyright 2010, Elsevier.

Given the discussion presented above, an interesting comparison of the voltammetric behaviour of quercetin and its 3-, and 4′-glucosides has been reported [72] on a glassy carbon electrode, in 0.1 M sodium acetate-acetic acid buffer in 90% methanol. For quercetin 3-glucoside, as expected, only two oxidation peaks were observed. The first reversible one corresponds to the oxidation of the *o*-dihydroxy moiety and the other one represents the irreversible oxidation of a hydroxyl group located in ring A. However, for quercetin 4′-glucoside, which does not have an *o*-dihydroxy moiety, three irreversible oxidation peaks were present. The first, whose potential was slightly shifted toward more positive values compared to quercetin 3-glucoside, was attributed to an electron oxidation of the hydroxyl group at C3′ to form the corresponding radical. Consequent one-electron oxidation of the hydroxyl group at C3, leading to the formation of a diradical, was proposed to be responsible for the formation of the second peak. The potential of the last peak is the same in both cases and is evidently related to the oxidation of a hydroxyl group in the A ring. Using the HPLC-DAD-ESI-MS technique, a 4′-glocoderivative of 1,3,7,8-tetrahydroxy-4*a*-methoxy-11*aH*-benzofuro[3,2-b]chromen-11-one (**IV**) was found as the main product of the bulk electrolysis of quercetin 4′-glucoside at a potential beyond the second peak. Scheme 8 presents the proposed mechanism for this transformation. The voltammetric behaviour of quercetin 4′,3-diglucoside has also been presented [73] under the same experimental conditions. Two irreversible peaks were observed. The latter is related to the oxidation of a hydroxyl group in ring A, the other occurred at the potential of the second peak observed for the oxidation of quercetin 4′-glucoside. Given the mechanisms discussed, this is an intriguing observation that deserves further investigation. 

The same authors [72,73,74,75,76] used electrochemical impedance spectroscopy to investigate the electrooxidation of quercetin, which is a unique approach. It has also been shown that quercetin undergoes electroabsorption on the electrode surface [74,75] and that a platinum electrode cannot be used for quercetin oxidation in methanolic media due to methanol electrooxidation at the same potentials [76].

Voltammetric curves measured on a glassy carbon electrode in 0.1 M sodium acetate-acetic acid buffer in 90% methanol have been presented [77] for oxidation of quercetin, as well as 6-*C*-, and 8-*C*-glucosides of luteolin and apigenin. From the point of view of the present discussion, the presence of the glucose substituents is not relevant. Quercetin and luteolin glucosides, which possess an *o*-dihydroxy moiety in ring B, show a reversible oxidation. Quercetin, which also has a hydroxyl group at C3, exhibits further irreversible oxidation at more positive potentials. Apigenin glucosides, which do not contain these moieties, are not electroactive in the potential region. All of the substances show the oxidation of a hydroxyl group in ring A. A similar observation has been reported for luteolin-8-*C*-glucoside (orientin) [78]. In this article, the voltammograms of eriodictyol (3′,4′,5,7-tetrahydroxyflavanone) and robinin (kaempferol 3-*O*-robinoside-7-*O*-rhamnoside) are presented. Orientin and eriodictyol, which possess a catechol moiety in ring B and do not have the C3-hydroxyl group, show reversible oxidation peaks at approximately +0.29 V and +0.25 V (vs. Ag/AgCl, when the solution was not indicated, it was not reported in the cited paper), respectively. Robinin, which does not have a catechol moiety in ring B and has a sugar moiety at C3, is irreversibly oxidized at approximately +0.75 V. Irreversible peaks at about +0.90 V, resulting from oxidation of a hydroxyl group in ring A, are present in all cases.

The cyclic voltammetric investigation of the electrochemical oxidation of quercetin on a glassy carbon electrode modified with multi-wall carbon nanotubes in aqueous 0.2 M phosphate solutions at different pH has shown the existence of three oxidation peaks [79]. The reversible oxidation at the first peak was described as an ECEC first-order process based on the comparison of the digitally simulated cyclic voltammetric curves with the experimental ones. This allowed to obtain the values of the kinetic parameters of the ECEC process. The electrochemical behaviour of quercetin, morin, and rutin was also studied [64] using cyclic, differential pulse, and square wave voltammetry methods on a glassy carbon electrode in acetate buffer at pH 3.6. As expected, the three peaks during the electrooxidation of quercetin were observed, while the second peak observed for quercetin was absent in the case of rutin and unexpectedly also for morin, which can be oxidized to the *p*-quinone methide and give the benzofuranoone derivative [66]. However, it is surprising that the potential values of the first peak for all compounds are very close in spite of the fact that morin does not have an o-dihydroxy moiety.

Finally, the redox properties of individual quercetin moieties have been characterized by the derivatization of particular quercetin hydroxyl groups [80]. The results presented obtained using electrochemical, structure-activity relationship studies and DFT (density functional theory) calculations confirm the mechanism presented in Scheme 6.

Myricetin is structurally similar to quercetin but differs by the existence of pyrogallol (3′,4′,5′-trihydroxy) moiety in ring B. Square wave voltammograms measured on paraffin-impregnated graphite electrode-covered by myricetin microcrystals in water media (pH 3) show the existence of two peaks at approximately +0.36 V (reversible) and +0.82 V (irreversible) vs. Ag/AgCl (3 M KCl) electrode [81]. However, the investigated scan range ended at +1.10 V. This is an important remark, since in the same article the voltammograms of dihydromyricetin (lack of carbon 2,3 double bond) were presented in the wider potential window and in addition to the two previously mentioned peaks an additional irreversible peak was observed at +1. 20 V. It is obvious that the first peak is caused by an *o*-quinone formation in ring B. The authors have claimed that the second and third peaks can be attributed to the oxidations of the phenolic and alcoholic hydroxyl groups, respectively, but no details on the mechanism have been provided. Taking into account the findings already described, it seems that the oxidation of the benzofuranone derivative, whose formation was reported in an independent study [66], causes the presence of the second peak, and the third peak results from the oxidation of a hydroxyl group in A ring. The presence of the second peak in the case of dihydromyricetin oxidation is somewhat surprising because the formation of the benzofuranone derivative requires the presence of a carbon 2,3 double bond (Scheme 7). However, the cyclic voltammograms of dihydromyricetin presented in articles related to its electrochemical determination [82,83,84] show a small anodic peak in the potential region.

**Scheme 9:**
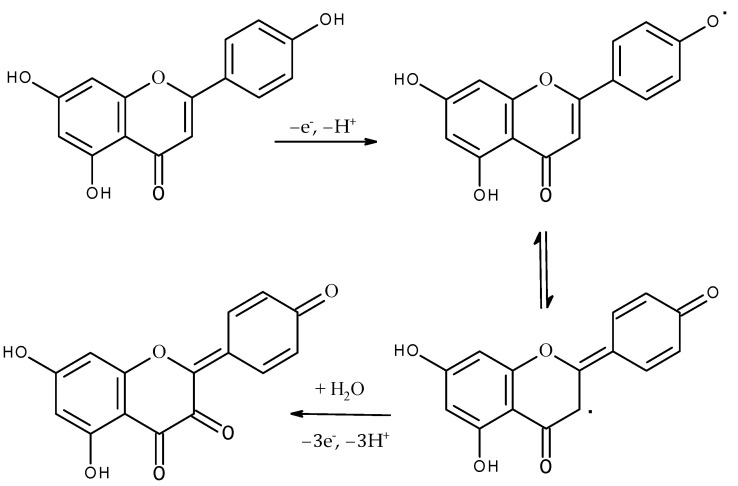
Proposed mechanism of electrooxidation of apigenin based on the experimental data presented in presented in Refs. [46,85].

Electrochemical oxidation of apigenin and acacetin (5,7-dihydroxy-4′-methoxyflavone) has been investigated on a glassy carbon electrode, in water-ethanolic solution by cyclic, differential pulse and square wave voltammetry [85]. At pH 4.3 apigenin shows two irreversible oxidation peaks at approximately +0.8 V and +1.0 V (vs. Ag/AgCl/3M KCl electrode), whereas, in the case of acacetin, only the peak at +1.0 v is present. For relatively slow scan rates (0.05 V s^−1^) the first peak of apigenin oxidation has the form of a broad shoulder, which is related to the adsorption of the substrate on the electrode surface. The authors postulated that hydroxylation of B and A rings occurs at the first and second potential, respectively. This proposition seems dubious because, at such positive potentials, *o*-dihydroxy moieties formed in both cases can be easily oxidized. The more realistic is the assumption, as previously postulated, that oxidation of a hydroxyl group in ring A occurs at more positive potential. The formation of the first peak in the case of apigenin oxidation is probably caused by the oxidation of the C4′ hydroxyl group and the allocation of an electron from the phenoxyl radical to ring C to form a carbon radical. The latter can be further oxidized to give *p*-quinone methide (Scheme 9). An analogous mechanism has been proposed for apigenin and 3-hydroxyflavone in nonaqueous media [46]. These results indicate that the hydroxylation of ring C or ring B in the case of apigenin and 3-hydroxyflavone, respectively leads to a configuration containing C3 and C4′ hydroxyl groups. At the same time, the presence of a C2–C3 double bond allows the formation of *p*-quinone methide. Acacetin, which contains a methoxy group at C4′ cannot form a *p*-quinone methide and consequently, the first peak is absent. However, it should be admitted that the corresponding benzofuranone derivative, as a consequence of the formation of *p*-quinone methides, has not been detected during the preparative electrolysis of apigenin [66].

The cyclic voltammogram of 3-hydroxyflavone on a glassy carbon electrode in a water-methanol matrix displays at pH 3 two irreversible peaks at approximately +0.45 V and +0.90 V (vs. Ag/AgCl, 3 M KCl) [86]. It has also been shown that the potential of the first peak measured by the differential pulse technique is shifted toward more positive potentials with the increase of pH. The proposed mechanism postulates the formation of a radical at pH 3 and a cation at pH 8. However, the formation of a carbon radical and then the *p*-quinone methide, as proposed for apigenin cannot be excluded. 

A similar conclusion can be drawn from the comparison of the electrochemical behaviour of isoflavones: genistein [85,87] (4′,5,7-trihydroxyisoflavone), daidzein [87] (4′,7-dihydroxyisoflavone), and biochanin A [87] (5,7-dihydroxy-4′-methoxyisoflavone). Biochanin A, in which the C4′ position is occupied by a methoxy group, in contrast to genistein and daidzein, does not exhibit the first peak. Another study [88] has compared the cyclic voltammograms of daidzein and 7-hydroxy-4-chromone, which differs from daidzein by the lack of ring B. As expected, the first peak does not appear in the case of 7-hydroxy-4-chromone. A mechanism assuming one-electron oxidation of the 4′-hydroxyl group and 7-hydroxyl group, leading to the formation of corresponding radicals and their dimerization or reaction with solvent, has been proposed to explain the formation of the first and the second peak, respectively.

Chrysin molecule contains only two hydroxyl groups in *meta* position (C5 and C7) in ring A. Cyclic voltammogram taken on a glassy carbon electrode in a water-based phosphate buffer at pH 7 exhibits an irreversible peak at approximately +1.0 V (vs. Ag/AgCl, sat. KCl) [41]. The oxidation of a hydroxyl group and eventual subsequent reactions of the oxidation product with reaction media are responsible for the peak formation. In contrast, baicalein (5,6,7-trihydroxyflavone), which has a pirogallol moiety in ring A shows a reversible oxidation peak at approximately +0.1 V (vs. Ag/AgCl) when oxidized on a glassy carbon electrode in a water buffered solution at pH 7.4 [89]. Similarly, 7,8-dihydroxyflavone exhibits a quasi-reversible oxidation peak at approximately +0.4 V (vs. Ag/AgCl) [90]. Clearly, the peaks can be related to the formation of corresponding *o*-quinones.

The data presented herein on the electrochemical behaviour of flavonoids have provided a general insight into the relationship between the structure of the flavonoids and their oxidation mechanism. However, these data were usually collected under different experimental conditions. Therefore, it was sometimes difficult to compare the results presented and provide an unequivocal interpretation. It is interesting to analyze the papers in which a series of flavonoids was investigated under the same experimental conditions. Unfortunately, some of the papers present data limited to the first oxidation potential.

The redox potentials of the first oxidation step for a series of flavonoids were determined by cyclic voltammetry in phosphate buffer containing 2.5% DMSO at pH 7.4 concerning the reduction of ferryl myoglobin [91]. The first oxidation potentials of a series of structurally related flavonoids have also been measured by cyclic voltammetry (on plastic-formed carbon) and flow-through column electrolysis (on carbon fibres) in phosphate buffer at pH 7.5 [92]. Good agreement was received between the potential values measured by both methods. It was shown that there is a relationship between the potential values obtained from the hydrodynamic voltammograms and the antioxidant data (IC_50_) in biological systems. The less positive oxidation potentials were generally associated with a decrease in IC_50_ values [93]. In a series of articles [94,95,96,97,98,99,100], the values of the first oxidation potentials of several flavonoids, measured by square wave voltammetry on a glassy carbon electrode in an aqueous medium at pH 3 and 7 have been reported. The values were used to verify the accuracy of the calculated potential values using a procedure developed by the authors. The procedure is based on a theoretical calculation of the sum of the differences in the net atomic charges on carbon atoms for different electronic structures of a flavonoid. However, knowledge of only the first oxidation potential value strongly limits the mechanistic considerations.

Cyclic voltammograms of the 14 flavonoids measured on a glassy carbon electrode in Britton-Robinson buffer containing KCl at pH 7.0 have been presented [59]. The voltammograms were registered between −0.4 V and +1.4 V (vs. Ag/AgCl), which allowed us to trace the possible oxidation beyond the first one and up to three oxidation peaks were visible in the potential window. Based on the peak potentials observed for quercetin, the oxidation potentials for other flavonoids were grouped in the corresponding regions. The data are presented in Table 1. There are no considerations of the mechanism of the oxidation process, the potential values have been correlated with antioxidant activity parameters, determined by different spectrometric assays. The discussion of the data presented in Table 1 is provided in Section 4.

**Table 1:** Comparison of the potentials of the oxidation peaks of flavonoids measured under the same experimental conditions in different solvents.

	Compund	Potential [59] [V, vs. Ag/AgCl] in Water			Potential [101] [V, vs. Ag/AgCl] in Ethanol		Potential [46] [V vs. SCE] in Acetonitrile		
		I	II	III	I	II	I	II	III
1	Quercetin 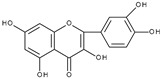	0.202	0.395	0.9 ^a^	0.65	1.0	0.83	1.12 and 1.22	1.68
2	Luteolin 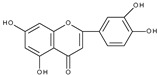	0.261	n.o.	1.021	0.71	n.o.	1.03	n.o.	1.68
3	Myricetin 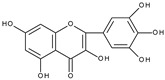	0.119	0.598	n.l.	0.54	1.02	0.79 ^b^	1.21	1.65
4	Kaempferol 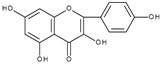	0.194	0.384	n.l.			n.o.	0.98	1.68
5	Fisetin 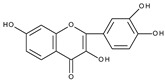						0.85	1.08 and 1.20	1.68
6	Taxifiolin 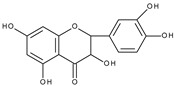	0.238	n.o.	0.966	0.86 (broad peak)	n.o.			
7	Ampelopsin 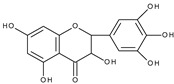				0.64	1.04			
8	Apigenin 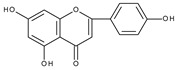	n.o.	0.623	0.821			n.o.	1.12	1.63
9	Naringenin 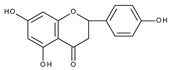	n.o.	0.710	0.955					
10	3-hydroxyflavone 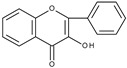						n.o.	1.10	n.o.
11	5-hydroxyflavone 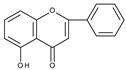						n.o.	n.o.	1.61
12	7-hydroxyflavone 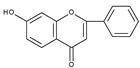								1.62
13	Chrisin 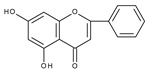				n.o.	n.o.	n.o.	n.o.	1.61
14	Rhamnetin 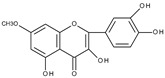				0.69	1.04			
15	Isorhamnetin 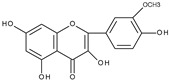	0.116	0.564	1.05 ^a^	0.71	1.14			
16	Quercitrin 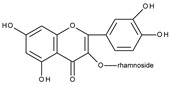	0.326	n.o.	0.960					
17	Hesperetin 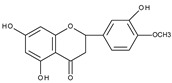		0.524	1.002					
18	Daidzein 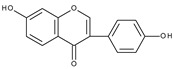		0.601	0.922					
19	Genistein 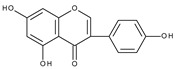		0.614	0.903					
20	(+) Catechin 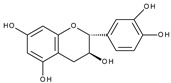	0.266	0.609	n.o.					
21	(−) Epicatechin 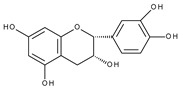	0.208	0.595	n.o.	0.76	1.02			
22	Galangin 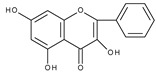				0.82	n.o.			
23	Gossypetin 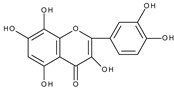				0.49 and 0.70	1.15			
24	Tamarixetin 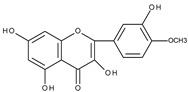				0.74	0.99			
25	Epigallocatechin 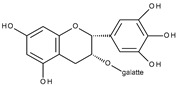				0.77	0.96			
26	7,8-dihydroxyflavone 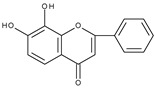						1.05 ^b^	n.o.	n.o.
27	Baicalein 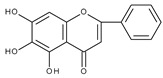						0.89	n.o.	1.59
28	3′,4′,3-trihydroxyflavone 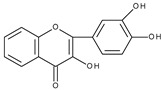						1.02	1.26 and 1.32	n.o.
29	Morin 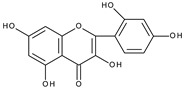						n.o.	0.97	1.7
30	Flavone 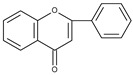						n.o.	n.o.	n.o.

^a^ the value was taken from the voltammogram published, ^b^ the peak shows a shoulder, n.o.—not observed. n.l.—the value of the potential was not listed and the voltammogram was not published, if the potential field does not contain any description, the CV was not reported.

## 3. The Electrochemistry of Flavonoids in Organic Media

There are much less papers, which describe the electrochemical behaviour of flavonoids in organic media. The purpose of the use of an organic solvent was to increase the solubility of investigated compounds and also to lower the reactivity of the radical intermediates, which can be formed during electrochemical processes.

Cyclic and differential pulse voltammograms of flavone, morin, rutin, quercetin, and apigenin on a platinum electrode in acetonitrile have been presented in [102]. For these compounds and also for chrisin and hesperidin (hesperetin 7-rutinoside, hesperetin: (2S)-3′,5,7-trihydroxy-4′-methoxyflavan-4-one) the values of the first oxidation potentials are listed and transition coefficients and heterogeneous rate constants were determined. Somewhat surprising is the presented voltammogram for flavone showing two oxidation peaks at potentials more positive than those for quercetin. On a glassy carbon electrode in acetonitrile flavone is not electroactive in the potential region, where quercetin shows three oxidation peaks [46]. In subsequent research [103], a mechanism of morin electrooxidation leading to *p*-quinone methide has been postulated.

**Scheme 10:**
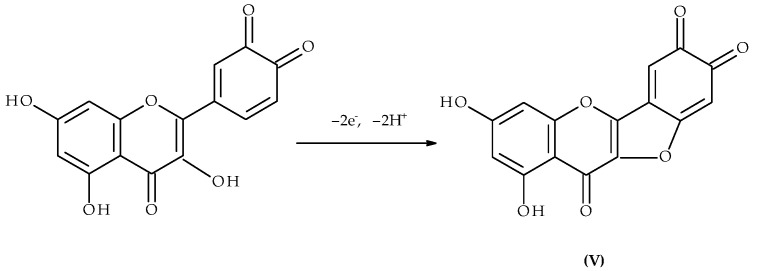
Proposed mechanism for the second peak formation during oxidation of quercetin on a glassy carbon electrode in DMSO. Adapted with permission from Ref. [104]. Copyright 2010, Elsevier.

The cyclic voltammogram of quercetin on a glassy carbon electrode in dimethyl sulfoxide (DMSO) recorded between −1.0 V and +1.5 V (vs. SCE) shows two irreversible oxidation peaks [104]. Based on the number of electrons consumed during controlled potential electrolysis at the potentials corresponding to the oxidation peaks and UV-vis spectra, it was suggested that the first step includes the oxidation of quercetin to the corresponding 3′,4′-quinone. Its further oxidation, originating at the C3 hydroxyl group leads to the formation of 1,3-dihydroxybenzofuro[3,2-b]chromene-7,8,11-trione (**V**) and the process is responsible for the formation of the second peak (Scheme 10). The proposed mechanism looked like a reasonable explanation for the second peak formation. However, it has been reported [50] that quercetin oxidized by bulk electrolysis in acetonitrile on a reticulated vitreous carbon electrode, at a potential of 0.2 V more positive than the half-peak potential determined by cyclic voltammetry, gives the benzenofuranone derivative (**II**) as the sole product. The authenticity of the product was confirmed by LC-MS and ^1^H- as well as ^13^C-NMR methods. During the bulk electrolysis of kaempferol and luteolin performed under the same conditions, the corresponding benzenofuranone derivative was detected only for kaempferol. In the case of luteolin, no oxidation products were found despite its consumption during electrolysis. Complete electrolysis required approximately 2 electrons per substrate molecule (1.9 for quercetin and 1.85 for both kaempferol and luteolin). Taking into account the structures of the flavonoids investigated, the results indicate that the lack of the C3 hydroxyl group in the case of luteolin prevents the formation of benzofuranone.

Cyclic voltammograms of quercetin recorded in acetonitrile on a glassy carbon electrode in the potential window from −0.5 to +1.6 V (vs. Ag/AgCl, 1 M LiCl) have shown the existence of two peaks [54]. It should be noted that other research performed in acetonitrile on glassy carbon [46] and platinum [105] electrodes in a wider potential window, reported the presence of the third oxidation peak at more positive potentials. The first peak forms as a result of the oxidation of quercetin to quercetin 3′,4′-quinone (Scheme 6). It has also been suggested that quercetin 3′,4′-quinone can be produced in a disproportionation reaction of radical anions, which are formed after the abstraction of an electron and two protons from a quercetin molecule. The oxidation of the quercetin benzofuranone derivative (**II**), formed in the reaction of quercetin 3′,4′-quinone with traces of water, has been proved as responsible for the existence of the second peak. The previously mentioned article [54] also presents cyclic voltammograms of luteolin (lack of the C3-hydroxyl group) and apigenin (lack of the C3′- and C3-hydroxyl groups compared to the quercetin molecule), which do not have peaks in the potential region where the oxidation of (**II**) occurs. Similarly, a cyclic voltammogram of taxifolin, taken under the same experimental conditions, does not show peaks in the potential region of (**II)** oxidation occurrence [106]. Taxifolin does not have the C2–C3 double bond compared to the quercetin molecule.

**Figure 2:**
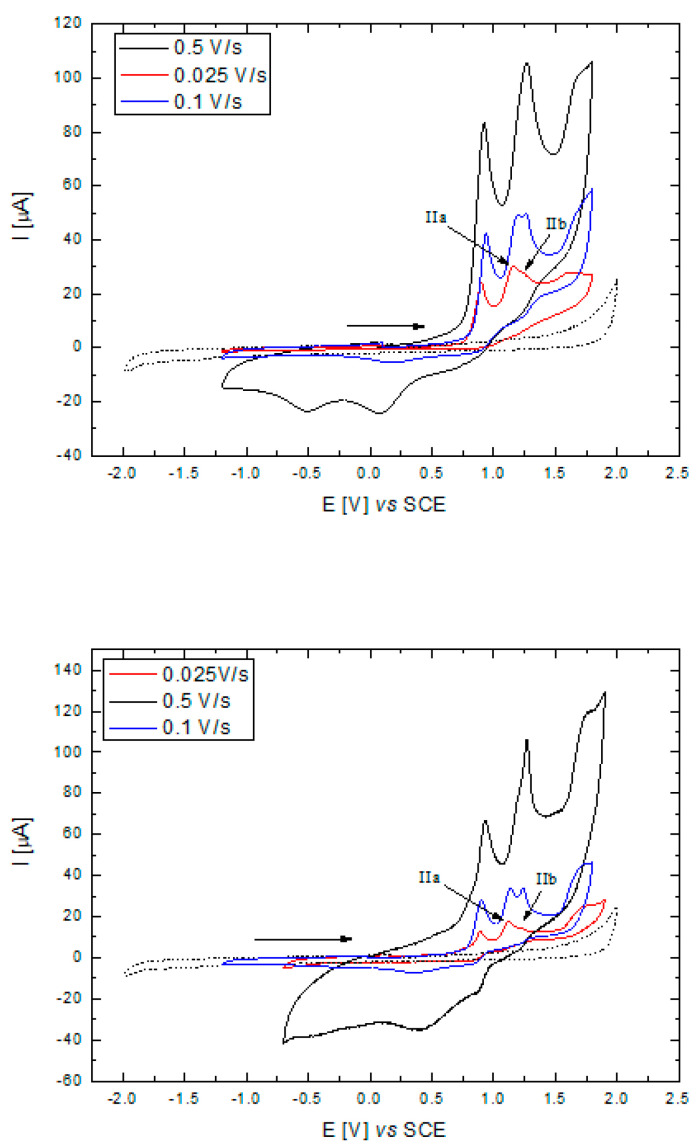
Cyclic voltammograms in MeCN [0.1 M LiClO_4_] for the: 1 mM quercetin (**top**) and 1 mM fisetin (**bottom**) in anodic scan registered first. Scan rate 0.025 V s^−1^ (red line), 0.1 Vs^−1^ (blue line), and 0.5 V s^−1^ (black line), supporting electrolyte (dashed line). (GCE (0.09 cm^2^; SCE vs. NHE, +0.242 V). Reproduced with permission from Ref. [46]. Copyright 2022, John Wiley and Sons.

It has been found [46] that for flavonoids, which have C3 and C4′ hydroxyl groups and also a hydroxyl group at the *ortho* position to C4′ (3′,4′,3-trihydroxyflavone, fisetin, and quercetin) two peaks are present in the second potential region at low scan rates, on cyclic voltammograms measured in acetonitrile on a glassy carbon electrode (Figure 2). The height of the prepeak (IIa, in Figure 2) decreases with increasing scan rate and at the scan rates usually above 0.1 V s^−1^, it disappears. The potential of the second peak (IIb), which is observed at higher scan rates as a single one, corresponds to the oxidation of the benzofuranone derivative. The appearance of the prepeak has been explained by the assumption that the products of the benzofuranone derivative oxidation easily adsorb on the electrode surface.

**Scheme 11:**
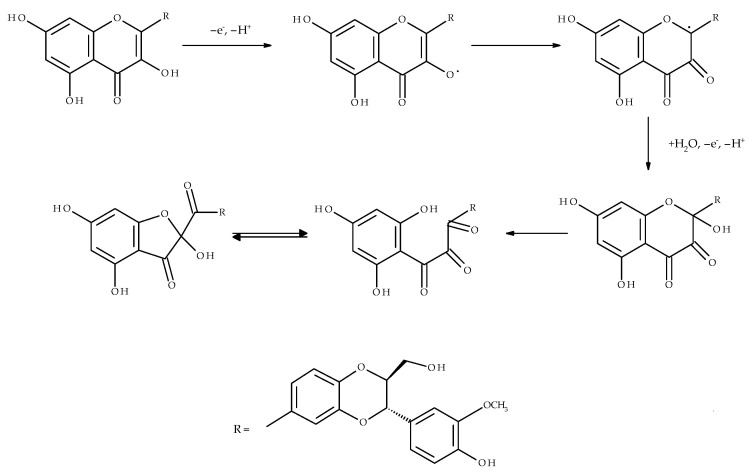
Mechanism for the formation of benzofuranone derivatives during the electrooxidation of 2,3-dehydrosilybin. Adapted with permission from Ref. [107]. Copyright 2017, American Chemical Society.

The data presented so far have indicated that the benzofuranone derivative can be formed when a flavonoid molecule contains C3 and C4′ hydroxyl groups as well as the C2–C3 double bond. However, the benzofuranone derivative has been shown to be produced during the electrooxidation of 2,3-dehydrosilybin [107], which molecule does not meet the conditions. The cyclic voltammogram of 2,3-dehydrosilybin recorded in acetonitrile on a glassy carbon electrode exhibits three oxidation peaks. The addition of water results in the shifting of the peak potentials toward less positive values. A more detailed mechanism than that proposed in [107] is presented in Scheme 11. The phenoxy radical formed at the first peak, after the removal of an electron and a proton, rearranges to the carbon radical, which is attacked by a water molecule. Further abstraction of an electron and a proton leads to the product—the benzofuranone derivative. Its electrooxidation results in the formation of the second peak. This statement was proved by the use of voltammetric and spectroelectrochemical data obtained for the authentic benzofuranone sample. The peak present at the most positive potential is due to the oxidation of a hydroxyl group present in ring A. Recorded under the same experimental conditions, the cyclic voltammogram of silybin, which differs from 2,3-dydrosilybin by the lack of C2–C3 double bond, has shown that the second peak is not present [108]. It was found that at the potential of the first peak, a silybin phenoxy radical was generated utilizing the hydroxyl group located next to the methoxy group, and then the hydroxylation, by traces of water, of the ring takes place. The sylbin radical was detected using in situ EPR spectroelectrochemistry in the presence of 5-tert-butoxycarbonyl-5-methyl-1-pyrroline-*N*-oxide (BMPO) as a spin trap reagent. The adduct of the silybin radical with BMPO was also identified using UPLC-ESI-MS/MS. The peak observed at the most positive potentials in both cases is caused by the oxidation of a hydroxyl group in ring A. The findings presented are in accordance with the possibility of hydroxylation of B or C rings during the electrooxidation of 3-hydroxyflavone and apigenin, respectively, suggested in [46] (Scheme 9).

An interesting comparison of the voltammetric behaviour of three series of flavonoids, which do not contain any substituents in ring B but have the hydroxyl group at position C5 in ring A, was presented for experiments carried out in acetonitrile on a platinum electrode [109]. For all flavonoids, only one oxidation potential has been indicated. For flavonoids, which do not contain the C2–C3 double bond—pinostrobin (5-hydroxy-7-methoxyflavanone), pinobanksin (3,5,7-trihydroxyflavan-4-one), and pinobanksin-3-acetate—the oxidation peak occurs at approximately +2 V (vs. SCE) regardless of the presence of the hydroxyl group at C3. When the C2–C3 double bond is present but there is no hydroxyl group in position 3—chrysin and tectochrysin (5-hydroxy-7-methoxyflavone)—the potential value is approximately equal to +1.9 V, while the presence of the C3 hydroxyl group—galangin and isalpinin (3,5-dihydroxy-7-methoxyflavone)—generates oxidation peaks at +1.54 V and +1.42 V, respectively. In the last case, the observed peaks probably result from the hydroxylation of ring B at the C4′ position and the subsequent formation of a corresponding *p*-quinone methide. If so, the peaks at approximately +2.0 V, caused by the oxidation of a hydroxyl group in ring A should be visible, however, the cyclic voltammograms for galangin and isalpinin have not been provided. In this study voltammograms, in which the cathodic scan was performed first, have been presented for chrysin and pinobanksin-3-acetate, and for other investigating flavonoids the values of the reduction potentials were listed. The investigated compounds exhibited two or three reduction peaks. Using in situ EPR spectroelectrochemistry, it has been shown that the oxidation and reduction processes originate with the formation of corresponding radicals as a result of one-electron transfer.

Cyclic voltammograms of kaempferol electrooxidation on pyrolytic graphite have been reported [110]. However, their interpretation is difficult because it looks like the voltammograms have started to register at the potential where a cathodic process has already occurred. Apigenin and naringenin (2,3-dihydroapigenin) covalently grafted on a glassy carbon electrode have been electroxidized in acetonitrile [111]. The presented voltammograms indicate that there is no substantial difference between them. This is a surprising result, which is not in accordance with the oxidation of analogous systems, for example, quercetin-taxifolin and 2,3-dehydrosilybin-silybin.

A comparison of the electrochemical behaviour of 13 flavonoids with their bond dissociation energies in relation to the antioxidant power has been presented in [101]. Cyclic voltammograms were recorded between −0.5 V and +1.5 V (vs. Ag/AgCl) on a glassy carbon electrode in ethanol (Table 1). It should be noted that the values of the oxidation potentials of flavonoids measured in ethanol are shifted toward more positive values compared to those in water and, therefore, the third peak visible in the water matrix was not recorded. The values of oxidation potentials and cyclic voltammograms measured on a glassy carbon electrode in acetonitrile for 15 flavonoids have been reported [46]. The potential values, grouped into three regions according to the values obtained for quercetin are listed in Table 1.

## 4. Discussion of the Observed Mechanisms of Flavonoids Electrooxidation

The data presented in Table 1 provide a convenient way to illustrate the reaction mechanisms suggested by comparing the oxidation potential values of the investigated flavonoids with those of quercetin, measured under the same experimental conditions.

**Scheme 12:**
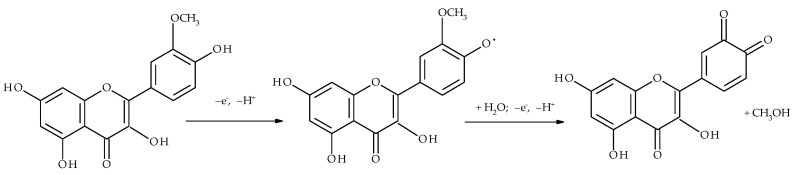
Putative mechanism of isorhamnetin electrooxidation proposed on the data available in Refs. [111,112].

In all cases, where an *ortho* dihydroxy moiety is present in ring B or A, the first oxidation potential shows the least positive value (first potential region). There are however some exceptions. Kaempferol, which does not obey the rule, in the water matrix has been reported to show a behaviour similar to quercetin (Entry 4, all entries mentioned in the Section concern Table 1). This observation is difficult to explain because in acetonitrile such behaviour is not observed and the cyclic voltammograms recorded on plastic-formed carbon electrodes in methanol-containing phosphate buffer show the shift of the first oxidation peak of kaempferol toward more positive values compared to quercetin [29]. Isorhamnetin (Entry 15) and tamarixetin (Entry 24), which have an *ortho* hydroxy-methoxy moiety in ring B, show the first oxidation peak in the first potential region. The *ortho* hydroxy-methoxy moiety is likely oxidized to *o*-quinone with simultaneous demethylation (Scheme 12). This mechanism has been proven for the electrooxidation of 2-methoxyphenol [112,113]. However, for hesperetin (Entry 17), the first oxidation potential is shifted toward a more positive value. An interesting case represents gossypetin (Entry 23), which molecule contains *ortho* dihydroxy moieties in ring B and ring A, and it looks like the two moieties are oxidized in the first potential region.

In the second potential region, a benzofuranone derivative (**II**) is oxidized. The derivative can be formed via *o*-quinone (Scheme 6). This path requires the presence of hydroxyl groups at C4′ and C3 as well as the presence of a C2–C3 double bond in a flavonoid molecule (Entries 1, 3, 5, 14, 15, 23, and 28). Luteolin and quercitrin, which do not have the available C3 hydroxyl group, do not show peaks in this potential region (Entries 2 and 16). In this potential region, flavonoids can be electrooxidized to *p*-quinone methide if C3 and C4′ hydroxyl groups and the C2–C3 double bond are present in their structure (Scheme 7). This class of compounds represents kaempferol and morin (Entries 4 and 29). However, if the C2–C3 double bond and only one hydroxyl group in ring B or ring C are present, hydroxylation of a ring, through the carbon radical, can also lead to the formation of *p*-quinone methide (Scheme 9). This mechanism is characteristic for 3-hydroxyflavone and apigenin (Entries 10 and 8) as well as for the other structurally related flavonoids (Entries 18, 19, and to some extent 22).

There are some inconsistencies in the discussed voltammograms. For example, taxifolin (Entry 6), in contrast to quercetin (Entry 1), does not have the C2–C3 double bond, and does not exhibit any peak in the disused potential region. The same structure differences exist between naringenin (Entry 9) and apigenin (Entry 8) as well as between ampelosin (Entry 7) and myricetin (Entry 3). For all of these flavonoids, a peak appears in the second potential region. An analogous observation was made for catechins (Entries 20 and 21). Based on available literature it is difficult to explain this behaviour. Definitely, the observed phenomena are worth further investigation.

The data in Table 1 confirm that the peak present in the third potential region is associated with the electrochemical oxidation of a hydroxyl group in ring A. Flavonoids that do not have hydroxyl groups in ring A do not exhibit any peak in this potential region. In the case of catechins, which contain hydroxyl groups in ring A, any oxidation peak is visible in the potential region (Entries 20 and 21). However, in water media, the third peak is frequently masked by water oxidation, especially when the flavonoid concentration is low.

Knowledge about the electrochemical behaviour of flavonoids is necessary to understand their antioxidant properties. However, some inconsistencies between the general rules and experimental observations have been indicated for some flavonoids. Therefore, the review can be a good starting point for further investigation of the electrochemical properties of flavonoids. The discrepancies indicated can be caused by differences in experimental conditions, but different paths of the electrochemical oxidation process cannot be excluded. Furthermore, it should be noted that the electrochemical parameters (*E_p_*, *I_p_*) established by cyclic voltammetry on a glassy carbon electrode for flavonoids and other compounds showing antioxidant properties depend on the cleaning procedure of the electrode when alumina is used to polish it [114]. It is also necessary to remember that the use of alcoholic media can lead to the formation of alkoxy derivatives.

## 5. Conclusions

The data presented on the voltammetric behaviour of flavonoids and the discussion of related mechanisms allow us to present the general processes that take place during the electrochemical oxidation of flavonoids.

1. Potential region I. If a flavonoid molecule contains an *ortho* dihydroxy moiety in either ring B or ring A, the moiety is oxidized, mostly reversibly, at the least positive potentials (the easiest oxidation) to the corresponding *o*-quinone. When *o*-quinone is formed in ring B and the molecule also contains the C2–C3 double bond and the C3 hydroxyl group, *o*-quinone exists in equilibrium with *p*-quinone methides (Scheme 3) and with water produces the benzofuranone derivative (Scheme 6).
2. Potential region II. In the region, the benzofuranone derivative is irreversibly oxidized. However, in this potential window flavonoids, which do not have an *ortho* dihydroxy moiety but possess C2–C3 double bond and hydroxyl groups in position C3 or C4′ or in both positions can form *p*-quinone methide.
3. Potential region III. At the most positive potential, the oxidation of a hydroxyl group located in ring A occurs if there is no other hydroxyl group in the ortho position. The process is irreversible, radicals formed can dimerize or react with the solvent components. The lack of hydroxyl groups in ring A results in the absence of any oxidation peak in the potential region.

## Data Availability

Data are available on request from the authors.

## Supplementary Materials

The following supporting information can be downloaded at: https://www.mdpi.com/article/10.3390/molecules28227618/s1, Table S1: Alphabetical list of names and structures of the flavonoids mentioned in the paper.
