# Supplementary material for: Electrochemistry of Flavonoids
Source: Molecules. 2023 Nov 16;28(22):7618. doi: 10.3390/molecules28227618 (PMC10674230; doi:10.3390/molecules28227618)
Supplement: Supplementary file 1 [file molecules-28-07618-s001.zip › molecules-2713112-supplementary.pdf]

**Table S1.** Alphabetical list of names and structures of the flavonoids mentioned in the paper.

| | Name | IUPAC name | Structure |
|---|---|---|---|
| 1 | Acacetin | 5,7-dihydroxy-4′-methoxyflavone | 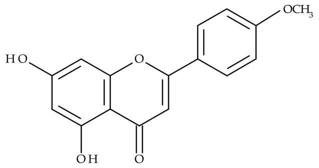 |
| 2 | Apigenin | 4′,5,7-trihydroxyflavone | 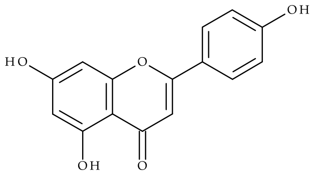 |
| 3 | Baicalein | 5,6,7-trihydroxyflavone | 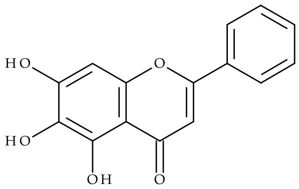 |
| **4** | Biochanin A | 5,7-dihydroxy-4′-methoxyisoflavone | 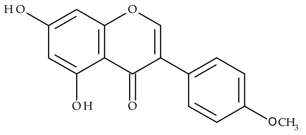 |
| 5 | (+) Catechin | *trans-3′*,4′,3,5,7-pentahydroxyflavane | 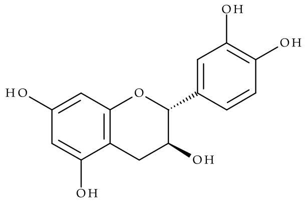 |
| 6 | Chrysin | 5,7-dihydroxyflavone | 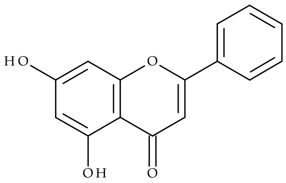 |
| 8 | Daidzein | 4′,7-dihydroxyisoflavone | 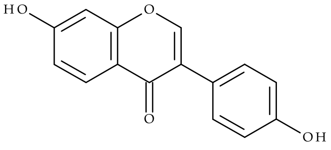 |
| 9 | | 2,3-dehydrosilybin | 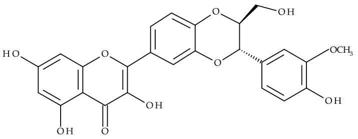 |
| 10 | | 7,8-dihydroxyflavone | 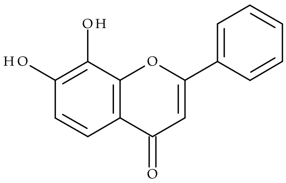 |

| | | | |
|---|---|---|---|
| 11 | (-)Epicatechin | *cis*-3′,4′,3,5,7-pentahydroxyflavane | 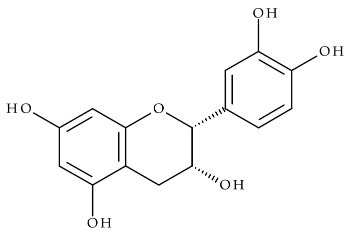 |
| 12 | Eriodictyol | 3′,4′,5,7-tetrahydroxyflavone | 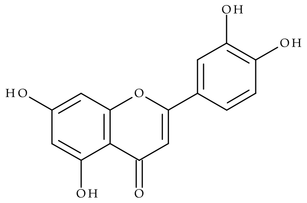 |
| 13 | Flavone | | 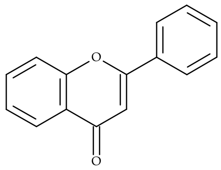 |
| 14 | Flavonol | 3-hydroksyflavone | 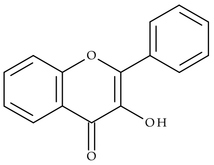 |
| 15 | Fisetin | 3′,4′,3,7-tetrahydroxyflavone | 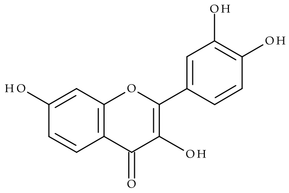 |
| 16 | Galangin | 3,5,7-trihydroksyflavone | 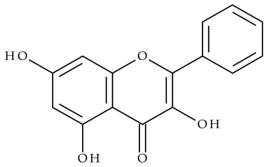 |
| 17 | Genistein | 4′,5,7-trihydroxyisoflavone | 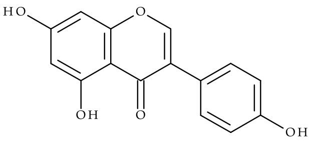 |
| 18 | Hesperidin | Hesperetin 7-rutinoside | 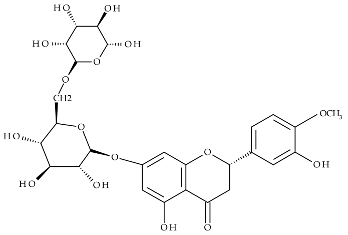 |
| 19 | Hesperetin | (2S)-3′,5,7-trihydroxy-4′-methoxyflavan-4-one | 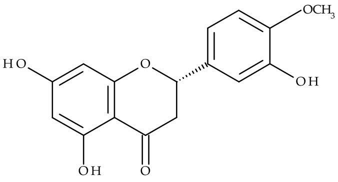 |

| | | | |
|---|---|---|---|
| 20 | | 7-hydroxyflavone | 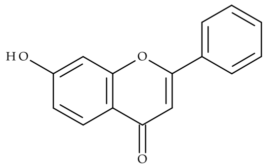 |
| 21 | Isalpinin | 3,5-dihydroxy-7-methoxyflavone | 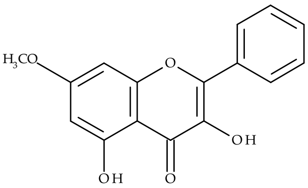 |
| 22 | Isorhamnetin | 4′,3,5,7-tetrahydroxy-3′-methoxyflavone | 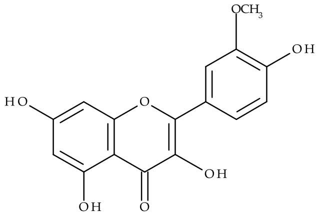 |
| 23 | Kaempferol | 4′,3,5,7-tetrahydroxyflavone | 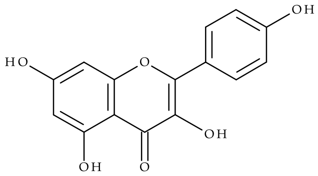 |
| 24 | Kaempferol 3-rutinoside | | 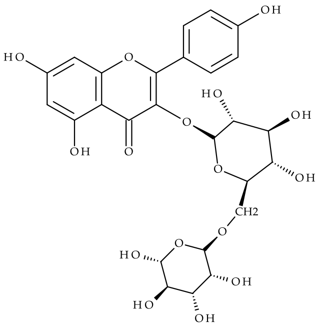 |
| 25 | Luteolin | 3′,4′,5,7-tetrahydroxyflavone | 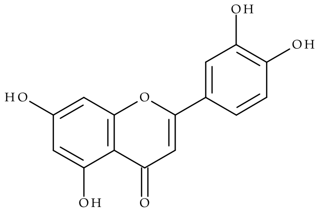 |
| 26 | Morin | 2′,4′,3,5,7-pentahydroxyflavone | 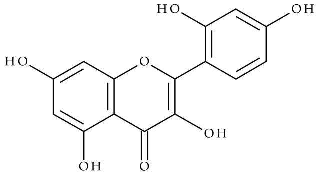 |
| 27 | Myricetin | 3′,4′,5′,3,5,7-hexahydroxyflavone | 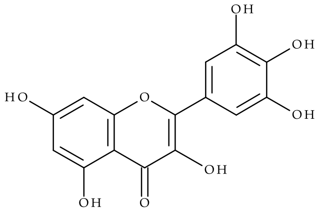 |

| | | | |
|---|---|---|---|
| 28 | Naringenin | 2,3-dihydroapigenin | 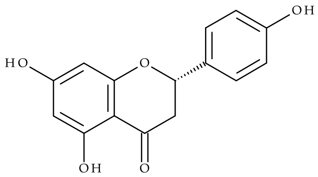  |
| 29 | Orientin | Luteolin-8-C-glucoside | 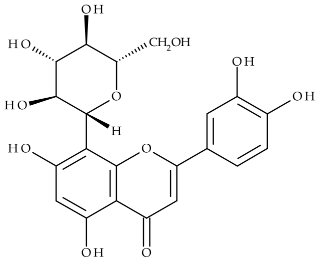  |
| 30 | Pinostrobin | 5-hydroxy-7-methoxyflavanone | 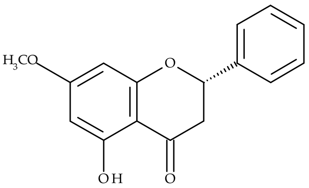  |
| 31 | Pinobanksin | 3,5,7-trihydroxyflavan-4-one | 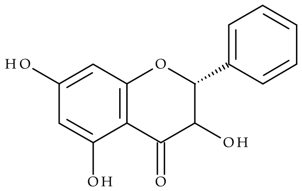  |
| 32 | Quercetin | 3′,4′,3,5,7-pentahydroxyflavone | 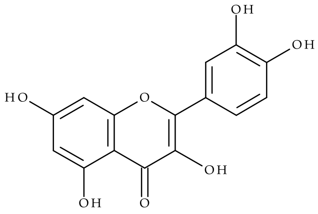  |
| 33 | Quercitrin | Quercetin-3-rhamnoside | 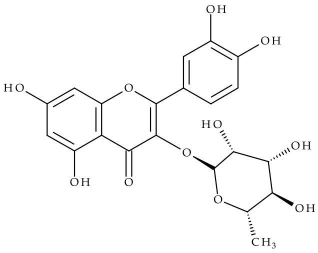  |
| 34 | Rhamnazin | 4′,3,5-trihydroxy-3′,7-dimethoxyflavone | 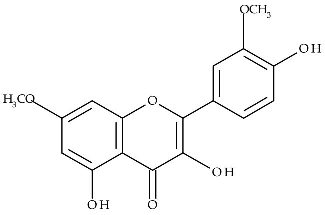  |
| 35 | Rhamnetin | 3′,4′,3,5-tetrahydroxy-7-methoxyflavone | 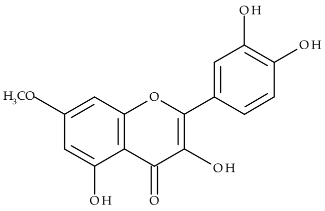  |

| | | | |
|---|---|---|---|
| 36 | Robinin | Kaempferol-3-O-robinoside-7-O-rhamnoside | 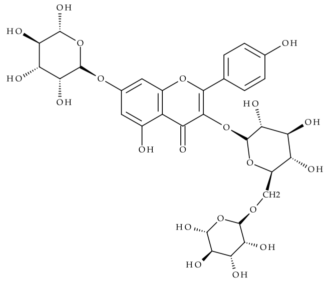 |
| 37 | Rutin | Quercetin 3-rutinoside | 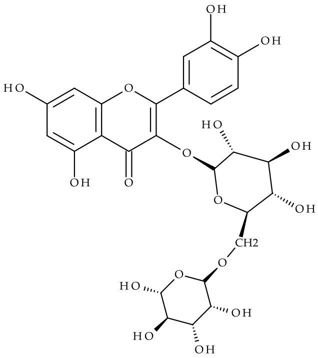 |
| 38 | Silybin | | 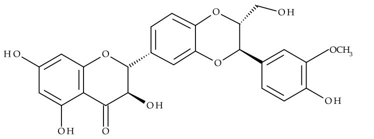 |
| 39 | Taxifolin | 3′,4′,3,5,7-pentahydroxyflavanone | 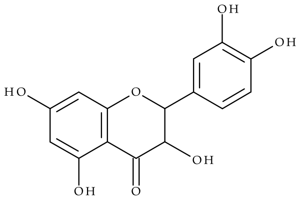 |
| 40 | Tectochrysin | 5-hydroxy-7-methoxyflavone | 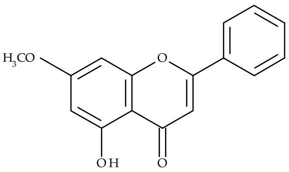 |
